# Latent Patient Cluster Discovery for Robust Future Forecasting and New-Patient Generalization

**DOI:** 10.1371/journal.pone.0162812

**Published:** 2016-09-16

**Authors:** Ting Qian, Aaron J. Masino

**Affiliations:** Department of Biomedical and Health Informatics, The Children’s Hospital of Philadelphia, Philadelphia, PA, United States of America; National Eye Institute, UNITED STATES

## Abstract

Commonly referred to as predictive modeling, the use of machine learning and statistical methods to improve healthcare outcomes has recently gained traction in biomedical informatics research. Given the vast opportunities enabled by large Electronic Health Records (EHR) data and powerful resources for conducting predictive modeling, we argue that it is yet crucial to first carefully examine the prediction task and then choose predictive methods accordingly. Specifically, we argue that there are at least three distinct prediction tasks that are often conflated in biomedical research: 1) data imputation, where a model fills in the missing values in a dataset, 2) future forecasting, where a model projects the development of a medical condition for a known patient based on existing observations, and 3) new-patient generalization, where a model transfers the knowledge learned from previously observed patients to newly encountered ones. Importantly, the latter two tasks—future forecasting and new-patient generalizations—tend to be more difficult than data imputation as they require predictions to be made on potentially out-of-sample data (i.e., data following a different predictable pattern from what has been learned by the model). Using hearing loss progression as an example, we investigate three regression models and show that the modeling of *latent* clusters is a robust method for addressing the more challenging prediction scenarios. Overall, our findings suggest that there exist significant differences between various kinds of prediction tasks and that it is important to evaluate the merits of a predictive model relative to the specific purpose of a prediction task.

## Introduction

Commonly referred to as predictive modeling, the use of machine learning and statistical methods to guide expectations of healthcare outcomes has been widely recognized as a key tool for supporting clinical decisions and promoting personalized medicine [1]. Recent examples include using a pattern aided logistic regression model to predict heart failure risk using EHR data [2], and applying the Latent Dirichlet Allocation topic models to the task of identifying patient safety events [3], clinical processes [4] as well as patient concerns [5]. The application of complex predictive models in these studies have led to novel data-driven insights that would have been difficult to obtain otherwise.

**Fig 1:**
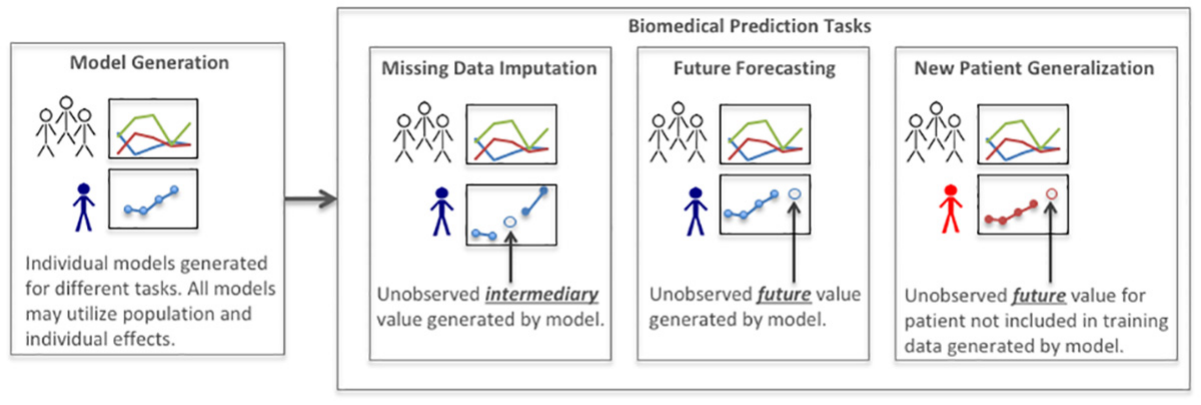
Predictive tasks in biomedicine. All models may utilize population and individual effects. Critically, for the missing data imputation and future forecasting tasks, models are trained on data that includes previous observations of the patient for whom predictions are made. In the new patient generalization task, the model is not trained using the patient’s observed data.

Given the general consensus on its usefulness, it is surprising that predictive modeling has received relatively little scrutiny as a formal method of biomedical informatics. Instead, the term “predictive modeling” has often been used as a general reference for any research involving the application of machine learning models, conflating the various *types* of prediction tasks. In fact, there are at least three distinct types of predictive tasks in biomedical informatics research—missing data imputation, future forecasting, and new-patient generalization (cf., [6]). Missing data are a common occurrence in many real world EHR datasets, due to factors such as non-responses and data input errors [7]. *Imputing missing data* is the process of “filling in” these unobserved values, which is often necessary for conducting further analyses as many machine learning and statistical procedures can only operate on so-called complete cases (i.e., variables of interest must all be observed for all subjects). *Future forecasting* is the process of using a model trained with previous observations for a given patient to project the development of a medical condition or any other trend for the same patient. Such results can be helpful in guiding expectations for patients and clinicians alike. Finally, *new-patient generalization* assesses the ability of a predictive model trained on previously observed patients to make predictions about newly encountered patients, such as in the case of using predictive models in a clinical setting. Moreover, generalization to new patients often occurs without retraining the predictive model, as there is usually no guarantee that sufficient resources or expertise is available for retraining in real-world applications. Fig 1 illustrates the differences between the three tasks.

The classification of predictive modeling into these three tasks highlights the differences in the goals and assumptions of a prediction task. For missing data imputation, the primary goal is to turn incomplete cases into complete cases for further analysis. There is a large body of literature on how this can be achieved with reasonable accuracy from a theoretical perspective [8, 9] and in applications to biomedical and epidemiological studies [10–13]. Although a detailed discussion of the issue of missing data itself (such as examining the origin of missing data) is beyond the scope of this paper, it is worth noting that in practice a common assumption is that the imputed data should follow the same statistical pattern as the observed data in order for the imputed cases not to bias further analysis (however, in some situations, the exact opposite might be true: the “missingness” of certain variables may signal systematic differences in patient populations; see [14] for an example). In other words, the unobserved missing data are assumed to be samples taken from the same distribution as the observed data.

However, this implicit assumption makes the term “prediction” technically a misnomer for the missing data imputation task, since the imputation process merely attempts to recover “masked” data that, by definition, conform to the already observed patterns. In contrast, the data to be predicted in both future forecasting and new-patient generalization are genuinely unobserved and are not necessarily, and should not be assumed to be, samples from the same distribution as the observed data. In the case of future forecasting, a patient’s underlying health condition may undergo unexpected changes, rendering factors of predictive value in the observed data less relevant. In the case of new-patient generalization, a predictive model may encounter new patients that exhibit health conditions that have not been observed before, where the predictive model must adapt to the new patient’s data in order to continue predicting successfully.

The uncertainty about whether the statistical patterns in newly encountered data deviate from the observed data is known as the problem of *concept drift* in the machine learning community [15–17]. A variety of techniques have been proposed to handle concept drift, including adaptive ensembles, instance weighting, and feature space adaption (for a review, see [17]). Although recent work in biomedical predictive modeling has started to *recognize* the issue of concept drift [6, 18], studies that specifically explore methods for handling the issue in biomedical data are few and far between (however, see [19] for an example). Our work here thus aims to accomplish two goals. First, we use regression-based predictive models and prediction of hearing loss progression as an example to demonstrate the various degrees to which concept drift affects data imputation, future forecasting, and new-patient generalization. Second, we propose the modeling of latent clusters as a robust method for handling concept drift in future forecasting and new-patient generalization.

### Handling Concept Drift via Latent Class Modeling

A seemingly straightforward solution to the problem of concept drift is continuously incorporating newly encountered observations into the training data, so that the predictive model can evolve with potentially changing patterns [20]. However, concept drift in healthcare data is rarely causally attributed to the passage of time. Instead, it is almost always driven by clinically and medically relevant factors. Each instance of concept drift usually signals a change in the underlying hidden context of a medical situation rather than occurring randomly (cf., [15]). Such changes in underlying contexts can occur not only over time (for example, as a result of changes in environmental factors) but also between different cohorts of patients observed at the same time. For instances, a shift in the effectiveness of an antibiotic drug could indicate newly developed antibiotic resistance [19] or variations in individuals’ reactions to treatment. Our strategy in finding a generalized method to deal with concept drift in healthcare data thus relies on inferring the underlying hidden contexts of data. Our approach focuses on mitigating the impact of potential concept drift in a prediction task by learning a *distribution* of hidden contexts from observed data. When concept drift occurs, we can then identify from the distribution of learned hidden contexts the one that best explains the currently observed data, and predict future observations based on that context only.

There are several advantages in adopting this approach. First, compared to many other fields, the data sets available in the healthcare domain are typically much larger in size (namely “big data”). For biomedical predictive modeling, the large volume of a typical EHR-derived data set makes it possible to discover a significant number of hidden contexts in the observed data, to such an extent that what appears to be concept drift in the newly encountered data is often merely a rehash of previously observed hidden contexts. In other words, with the abundance of healthcare big data, the chance of encountering a completely novel hidden context is dramatically reduced, making the problem of concept drift much easier as “drifting” occurs mostly between known hidden contexts. Second, learning a distribution of hidden contexts from observed data is a well-studied machine learning problem, which can be readily tackled by a variety of *latent class models*. In a latent class model, the observed data are assumed to be generated from a set of latent (i.e., hidden and unobservable) classes, and each data point is assumed to belong to a single class. Given the observed data, probabilistic inferences are performed on the parameters of each latent class as well as assignment of each data point to a latent class. In a prediction task, the inferred latent classes can then serve as the hidden contexts on which future observations are predicted.

Despite the fact that a variety of latent class models have been proposed in the machine learning literature, ranging from basic finite mixture models [21] to hierarchical [22] and infinite variants [23], there have been only limited studies that apply latent class models to healthcare data [3–5, 24]. Our current work thus represents one of the first investigations in this direction. We used a latent class model known as *mixture of regressions* to predict patterns of hearing loss progression in pediatric patients. To highlight its robustness in the face of concept drift, the mixture of regressions model is compared to two commonly used regression methods across three different prediction tasks introduced above: data imputation, future forecasting, and new-patient generalization.

## Methods

### Data

We obtained data from the Audiological and Genetic Database (AudGenDB), a public, de-identified research database derived from electronic health records (EHR) [1]. At the time of this study, AudGenDB contained data on more than 95,000 patients and audiograms of more than 78,000 patients. Overall, AudGenDB is intended to be a data resource that represents a population enriched for hearing loss research.

Audiogram test results in AudGenDB are recorded as the thresholds (in dB) at which patients respond to test stimuli at just above the chance level. We applied the following inclusion and exclusion criteria in our analysis: (1) Only audiogram tests documenting the specific ear tested (‘left’, ‘right’, or ‘soundfield’) are included; (2) Only bone-conduction tests are included; (3) Tests outside the frequency range of interest (500Hz, 1KHz, 2KHz, and 4KHz) were excluded; (4) Response thresholds recorded as 0 decibels were removed as they typically indicate a failed test attempt; (5) Patients with fewer than 4 tests across all frequencies of interest were also excluded in order to enable a 4-fold cross-validation procedure (described in more detail below); (6) Finally, test results recorded outside the age range of 0 to 21 were also removed in order to focus on a pediatric population.

Each individual ear of a patient at a given test frequency is coded as a separate entity, referred to as a “patient-ear-frequency” (PEF). This is done to account for the possibility that a patient may undergo hearing changes in each ear independently and hearing changes often differ arbitrarily across frequencies.

After applying these criteria, our bone-conduction dataset contains 53,585 test results of 3,446 unique PEFs. The number of test results per PEF ranges from 4 to 21, with a mean of 6 test results.

### Predictive models

We implemented three regression-based models for predicting hearing change patterns in our dataset. While regression models are typically used to estimate the correlations between an outcome variable and predictors of interest, a trained regression model can also function as a predictive model for new data (where the values of the outcome variable are predicted given the observed predictors). It is also important to clarify that these regression models are not meant to capture hearing change patterns in every detail. Instead, the goal is to create an accessible framework under which we can examine how concept drift affects the predictive capabilities of models with and without latent classes across different types of tasks. To this end, all regression models presented here implement the same basic regression form: to predict the threshold of an audiogram test given only the (log-transformed) age at the time of the test, the gender of the patient (dummy coded), and the (log-transformed) cumulative number of ear-related diagnoses at the time of that audiogram test (referred to as diagnosis below). Since our focus is on studying the *progression* of hearing changes, the primary predictor of interest is logarithmic age, while the other two predictors serve as control variables. Below, we first introduce the two regression methods without latent classes, and then describe how the mixture of regressions model differs from them by incorporating the learning of latent classes.

#### Ordinary Least Squares regression

We first consider the ordinary least squares regression (OLS) model with normally distributed residual errors (model fitting is performed by the lm function in R [25]):

$$threshold∼N(\beta \left[\begin{array}{c} 1\\ log(age)\\ gender\\ log(diagnosis) \end{array}\right],\sigma^{2})$$ (1)

where *β* is a vector that represents the regression coefficients, and *σ*^2^ is the residual variance. The OLS model produces a single set of regression coefficients, which we shall refer to as population-level effects. Essentially, the OLS regression implies that the progression of hearing loss as reflected in our data can be predicted by a single pattern: for *every* patient, their hearing loss progression can be predicted by the same set of coefficients describing the effects of their age, gender, and the number of ear-related diagnoses.

#### Mixed-effects regression

The second model that we consider is the mixed-effects regression, which includes both population-level fixed effects similar to the OLS model and individual-level (i.e., specific to each PEF) random effects, which are intended to better capture PEF-specific variations that may not be safely regarded as the residuals of the overall model. In addition to the same predictors as in the plain linear regression, the mixed-effects regression adds PEF-specific random intercept as well as random slopes for all predictors (model fitting is performed by the lmer function in R package lme4 [26]):

$$\left(threshold|{\text{PEF}}_{k}\right)∼N\left(\beta \left[\begin{array}{c} 1\\ log(age)\\ gender\\ log(diagnosis) \end{array}\right]+B_{k}\left[\begin{array}{c} 1\\ log(age)\\ gender\\ log(diagnosis) \end{array}\right],\sigma^{2}\right)$$ (2)

and

B∼N(0,Σ) (3)

Similar to the OLS regression, the mixed-effects regression assumes the presence of a central pattern of hearing loss progression in the data (captured by the fixed effects). Unlike the OLS regression, however, the mixed-effects regression allows each individual PEF to exhibit its own deviations from this central pattern (captured by the random intercept and slopes, as shown in Eq (3)). Importantly, in the context of a predictive task, the threshold predicted for a given PEF *k* will be based on not only the fixed-effect coefficients *β* but also the random-effect coefficients ***B***_***k***_ that are specific to PEF *k* (if *k* has been observed in the training data). As a result, the predictions of the mixed-effects regression are likely more accurate than the OLS regression in most cases.

#### The latent class model: Mixture of regressions

Finally, we consider a mixture of regressions model. We propose this model as a robust solution to the problem of concept drift in large healthcare datasets. At the center of the mixture of regressions is a latent clustering approach, which differentiates itself from the two alternative models introduced above. Specifically, the mixture of regressions model assumes that there are *K* *latent* clusters in the data, all of which follow the same regression form, but vary in the coefficient estimates of the predictors. In the case of our study, this means that each unique cluster specifies a different effect of how threshold can be predicted based on age, gender, and ear-related diagnoses. Furthermore, we impose the restriction that data points of the same PEF must belong to the same cluster, so that the data points of a single PEF is always described by one set of regression coefficients (model fitting is performed by R package flexmix [27]).

Our mixture of regressions model can be formally expressed as:

$$\left(threshold|{cluster}_{i}=k\right)∼N\left(\beta_{k}\left[\begin{array}{c} 1\\ log(age)\\ gender\\ log(diagnosis) \end{array}\right],\sigma_{k}^{2}\right)$$ (4)

and

$${cluster}_{i}∼\text{Multinomial}\left(\theta_{i}\right)$$ (5)

fori∈PEFs,andk∈1…Kclusters

The crucial assumption behind the mixture of regressions model is that there is potentially more than one common pattern of hearing loss progression in our data, each characterizing a different latent class of patients. For instance, some sub-populations in our dataset may have exhibited hearing *improvement* spanning the course of their hospital visits; other patients may have exhibited hearing loss progression at a rate much faster than the rest of the patients. These different groups of patients should belong to different latent classes. The mixture of regressions model is capable of accounting for these sub-populations in the data, if they exist, by categorizing cases showing similar patterns into common clusters. Contrary to the mixed-effects regression model, deviations within the same cluster are not modeled with dedicated parameters. Hearing thresholds for PEFs in the same cluster are predicted based on a common set of regression parameters instead (see Eq (4)). In this sense, the mixture of regressions model is a balanced approach that relaxes the assumption of only one central pattern in the OLS regression, but at the same time, imposes a more restrictive assumption than the mixed-effects regression.

Training a mixture of regressions model involves estimating both the probability distribution of each PEF’s cluster membership, and the *K* sets of regression coefficients, where *K* is specified manually (there are methods for inferring *K* automatically from the data [23], but a detailed treatment of that topic is beyond the scope of this article). In this work, we determined the optimal *K* by increasing the number of clusters until prediction accuracy on the withheld data reached asymptotic performance (more details below).

A potentially significant advantage of modeling latent clusters is the ability of a mixture of regressions model to flexibly use the clusters of sub-populations inferred during the training phase. For example, when a new audiogram result is to be predicted for a known PEF, the mixture of regressions model can readily retrieve the probability distribution of the PEF’s cluster membership *p*(cluster_*i*_ | *PEF*), and then predict the audiogram result by weighting the predictions of each cluster *f*_*k*_(age) by the probability of the PEF belonging to that cluster:

$$predicted\;threshold=\sum_{k}f_{k}\left(age\right)p\left({cluster}_{i}=k|PEF\right)$$ (6)

Even when the new audiogram result to be predicted comes from a new PEF, the mixture of regressions model can still infer which of the learned clusters the new PEF will *most likely* belong to as a first step. That is, the model is capable of incrementally revising its inference on *p*(cluster_*i*_|*y*_*i*1_, …, *y*_*in*_) for a new patient *i* as *n* gradually increases, by using the Bayes rule:

$$p\left({cluster}_{i}=k|PEF_{i}\right)\propto L\left(y_{i1},\ldots ,y_{in}|{cluster}_{i}\right)$$ (7)

The result of this inferred cluster membership can then be used in Eq (6). Such a mechanism requires no re-training of the model, as the inference of cluster membership for new PEFs is entirely based on parameters learned from the training data. As a result, we expect the mixture of regressions model to be particularly effective in the task of predicting audiogram results for new patients.

## Results

To examine the effect of concept drift on the above regression models in a predictive context, we created three simulated prediction tasks—a missing data imputation task, a future forecasting task (for existing patients), and a new-patient prediction task. In all three tasks, the predictive models were first trained on a training dataset, and then performed predictions on a testing dataset (the methods for selecting training and testing data are described below). These prediction tasks are “simulated” in the sense that the data being predicted are actually observed in the AudGenDB data set, but are purposefully excluded from the training data. Such a setup makes it possible to objectively evaluate the accuracy of model predictions by comparing predictions to actual observations.

### Task 1: Missing data imputation

To simulate the missing data imputation task, data points of each PEF were *randomly* split into 4 equally-sized bins (the above-mentioned exclusion criterion to exclude PEFs with at least 4 audiogram test results ensures that each bin has at least 1 data point). At any given time, three of the 4 bins were considered “observed”, while the remaining bin was considered as if its data were “missing” and needed to be predicted. Therefore, the goal of the first task can be conceived as data imputation, where the regression models must fill in the missing data.

Our method for evaluating the quality of data imputation is identical to a 4-fold cross-validation procedure. Across four iterations, the four bins of each PEF were rotated between serving as observed and missing data so that each bin was treated as the missing data exactly once. The three observed bins of all PEFs were aggregated and used to train each regression model. The trained regression models then predicted the hearing thresholds for each audiogram test in the missing data set. These predicted thresholds (i.e., the imputated values) were compared to the actual values, based on which the accuracy of prediction was evaluated using the mean squared error (MSE):

$$E_{i}=\frac{{\left(\hat{t}-t\right)}^{2}}{n_{i}},\;\text{for}\;i\in \;\text{all}\;\text{iterations}$$ (8)

where $\hat{t}$ is the predicted threshold of an audiogram test, *t* the observed threshold, and *n*_*i*_ the number of audiogram tests in the missing data set of iteration *i*. An average MSE score is then calculated for each model:

$$E=\frac{1}{4}\sum_{i}E_{i}$$ (9)

**Fig 2:**
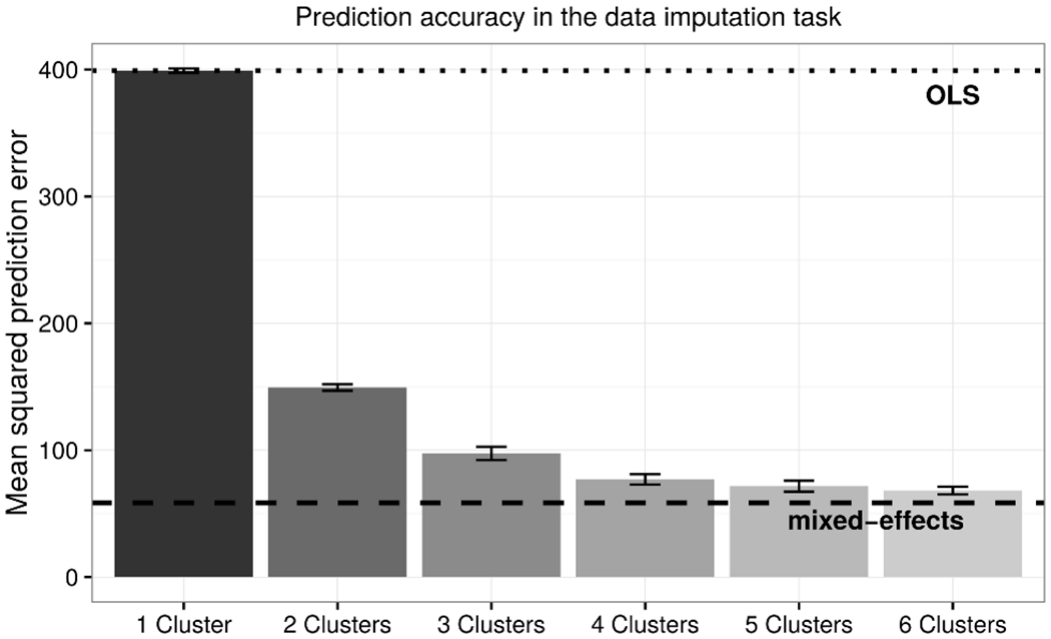
In the simulated data imputation task, the mixed-effects regression achieved the lowest (best) cross-validated average mean squared prediction error (MSE). As the number of clusters increases in the mixture of regressions model, the prediction error decreases dramatically and approaches that of the mixed-effects model. Error bar indicates between-fold standard deviation.

Fig 2 shows the average MSE of each regression model in this data imputation task. The average MSE score of the OLS model, shown by the dotted line, is the highest among all models at 399. This means that on average the predicted thresholds are approximately 20 decibels away from the actual observed values. In comparison, the mixed-effects regression (the dashed line in Fig 2) achieved an MSE of 59, implying that it can predict the response threshold of an audiogram test with a margin of error of 7.7 decibels. The MSE of the mixture of regression model decreases as the number of latent clusters is increased. When the number of latent clusters is 1, which reduces the mixture model to the OLS model, the MSE is equal to that of the OLS model. With the number of latent clusters set to 6, the mixture of regressions model achieves an MSE of 68.3, corresponding to an error of 8.3 decibels. Increasing the model complexity beyond 6 latent clusters results in extremely small clusters consisting of less than 1% of all PEFs (a threshold that we set heuristically), which suggests the presence of overfitting. Furthermore, in models where seven or more clusters were fitted by lifting this restriction on cluster size, no further improvement in accuracy was observed on the testing dataset (presumably because the added clusters were so small that their effects were negligible).

A striking finding in the current data imputation task is that the mixed-effects model has produced a dramatically better prediction accuracy than the OLS model. This large improvement in prediction accuracy not only indicates the practical usefulness of the mixed-effects model, but also reveals in what form concept drift might be present in our data. Since the mixed-effects model adjusts to the individual hearing change patterns that may exist in each individual (i.e., each PEFs in the context of our study), the improved accuracy suggests that there exist large variations between the hearing change patterns of the individual PEFs. In other words, concept drift occurs *between individuals* to a significant extent in our data. This interpretation is further corroborated by the regression coefficients. As shown in Table 1, the correlation between response threshold and age is not statistically significant except for one of the four folds under the OLS model. Furthermore, all OLS models found a significant negative correlation between hearing threshold and the cumulative number of diagnoses. Such an effect is counter-intuitive as a *general* pattern among patients, as more ear-related diagnoses should be correlated with an increase in threshold (i.e., hearing loss) rather than a decrease. In comparison, the mixed-effects regression provides population-level fixed effects that are much more intuitive: across the four folds, response threshold is consistently positively correlated with age, suggesting a hearing *loss* pattern in general. Importantly, the number of diagnoses is now positively correlated with threshold across all folds, suggesting that, on average, patients with more ear-related diagnoses will experience progressively worse hearing. By accounting for between-individual concept drift with random effects, the mixed-effects regression not only led to a much higher prediction accuracy than the OLS regression, but also yielded much more interpretable estimates.

**Table 1:** Correlation between audiogram response threshold and predictors as estimated by the OLS model and the linear mixed-effects regression model (only population-level fixed effects are shown). Numbers in parentheses indicate standard errors.

	*OLS*				*linear mixed-effects (population-level)*			
	Fold 1	Fold 2	Fold 3	Fold 4	Fold 1	Fold 2	Fold 3	Fold 4
log(age)	0.43	0.59**	0.32	0.20	0.57*	0.48*	0.49*	0.77**
	(0.22)	(0.21)	(0.21)	(0.21)	(0.25)	(0.24)	(0.24)	(0.25)
gender(F)	0.41***	0.38***	0.39***	0.42***	0.48*	0.44*	0.42*	0.49*
	(0.1)	(0.1)	(0.1)	(0.1)	(0.2)	(0.2)	(0.2)	(0.2)
log(diag.)	-2.09***	-1.92***	-1.86***	-1.76***	0.39***	0.51***	0.41***	0.45***
	(0.09)	(0.09)	(0.09)	(0.09)	(0.07)	(0.07)	(0.07)	(0.07)
(intercept)	28.51***	28.01***	28.55***	28.74***	27.65***	27.82***	27.82***	27.24***
	(0.50)	(0.48)	(0.47)	(0.47)	(0.58)	(0.56)	(0.56)	(0.58)

Note:

*p<0.05;

**p<0.01;

***p<0.001

Given the indication of between-individual concept drift, it is then intuitive to understand why the mixture of regressions model was able to achieve a prediction accuracy approaching that of the mixed-effects regression. Examining the estimated regression coefficients of each cluster from one cross-validation fold in Table 2, we note that the mixture of regressions model discovered six distinctive hearing change patterns, each with a different initial hearing loss severity (i.e., the intercept), the progression rate (i.e., the coefficient of logarithmic age), and the effects of gender and the cumulative number of diagnoses. The presence of these different patterns suggests that although it is possible to cater to between-individual variations with individual-specific coefficient adjustments, such as with random effects in the mixed-effects model, there is enough consistency between sub-populations of individuals so that categorizing these variations into six general patterns (i.e., latent clusters) works almost equally well. Compared to the mixed-effects model, we observe a similar effect for the cumulative number of diagnoses that is also generally positive, but a much wider range of gender effects. Crucially, these inferred latent clusters revealed a novel and unexpected insight on our data: out of the six clusters of PEFs, only three showed significant hearing *loss*—i.e., a statistically significant positive correlation between response threshold and age, comprising approximately half of the total data. The other half of the data are split between two clusters with a robust *negative* correlation indicating hearing *improvement* over time, and another showing no significant correlation at all. The existence of sub-populations with seemingly healthy hearing or hearing improvement is unexpected, as AudgenDB is intended to be enriched for patients with progressive hearing loss (note patients with cochlear implants were removed according to our data preparation procedure). We note that clinically it is highly unlikely that the patients in these identified sub-populations actually experienced hearing improvement, rather that there was some data artifact that accounts for this observation. The identification of such spurious data is another potential benefit of the mixture of regressions model.

**Table 2:** Approximately only half of our data are exhibit a hearing loss trend, as shown by the coefficients of regression predictors in one sample cross-validation fold as inferred by the the mixture of regressions model. Clusters are reordered by the coefficient of age for better presentation. Numbers in parentheses indicate standard errors.

cluster size	Cluster 1	Cluster 2	Cluster 3	Cluster 4	Cluster 5	Cluster 6
	4,812	6,933	6,121	9,955	5,314	6,979
log(age)	2.73***	2.53***	1.12***	-0.25	-1.18***	-1.50***
	(0.37)	(0.39)	(0.33)	(0.22)	(0.18)	(0.49)
gender(F)	-18.73***	-16.76***	38.09***	-0.44	-30.27***	16.31***
	(0.53)	(0.53)	(0.53)	(0.30)	(0.44)	(0.33)
log(diag.)	0.36***	0.59***	0.30*	0.20**	-0.02	0.24***
	(0.17)	(0.17)	(0.14)	(0.07)	(0.07)	(0.06)
(intercept)	61.24***	44.02***	25.56***	16.74***	42.21***	12.58***
	(0.97)	(1.07)	(0.85)	(0.56)	(0.41)	(0.49)

Note:

*p<0.05;

**p<0.01;

***p<0.001

Overall, the results in the missing data imputation task have painted an interesting and yet mixed picture of hearing change patterns in our data: the mixed-effects regression has undoubtedly achieved the minimal prediction errors by modeling both population-level fixed effects and PEF-level random effects. However, its population-level fixed effects are potentially of limited value in describing the manner in which hearing change patterns are distributed in the dataset. For example, one may be tempted to interpret the coefficients of the mixed-effects regression (see Table 1) to indicate the existence of a central hearing *loss* pattern in our data, supplemented by PEF-specific deviations modeled via random effects. But such “deviations”, as the results of the mixture of regressions model show, constitute approximately half of the data. The mixture of regressions model has instead provided an interpretation based on latent clusters, where these “deviations” are grouped into common sub-populations that presumably experienced healthy hearing or hearing improvement. In the next two prediction tasks, we examine which of these two interpretations of the structure of the data is more appropriate, and consequently, which of the corresponding regression models has a more robust prediction mechanism.

### Task 2: Future Forecasting

In the second task, each PEF’s last audiogram was withheld to create the testing dataset, which corresponds to approximately 18% of total data. The regression models were thus trained on a chronologically consecutive sequence of audiogram results prior to the withheld data point for each PEF. This task mimics a scenario of predicting future outcomes of a patient based on *existing* history of the same patient, as the test set consists of observations that occur in the “future” from the perspective of a trained regression model.

**Fig 3:**
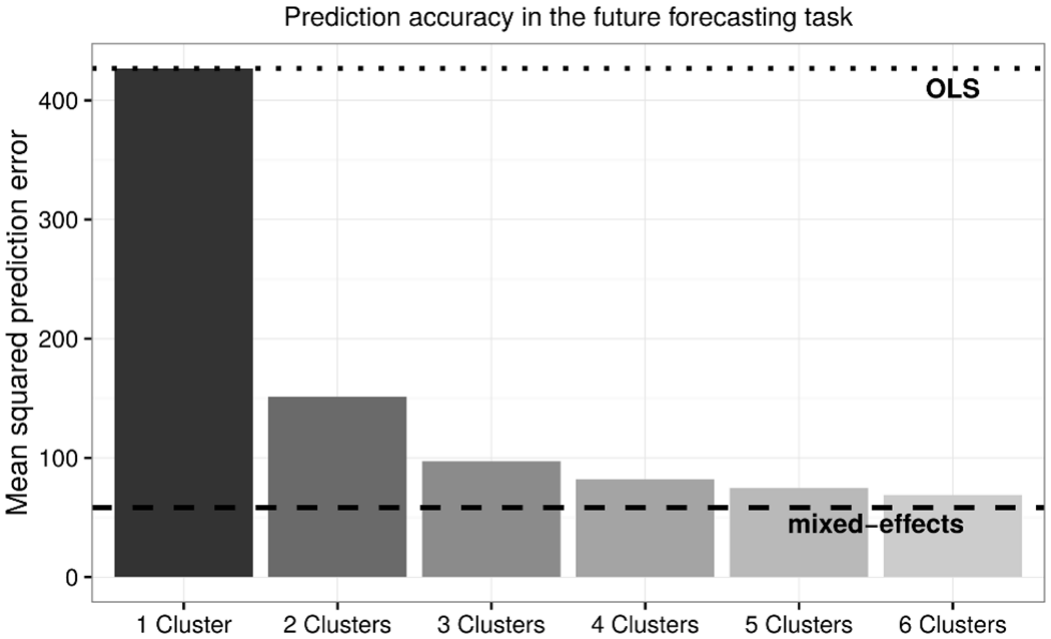
In the simulated future forecasting task, a pattern similar to the data imputation task is observed for average prediction error. The mixed-effects regression achieved the lowest MSE, and the mixture of regressions model approaches that accuracy level with five clusters.

In extrapolating observed data to future outcomes, it is possible that the previous hearing change pattern of a patient may no longer apply to the future time period, causing *within*-individual concept drift. For example, if a PEF showed no signs of hearing loss in the time period covered in the training set, but then experienced rapid hearing loss later on, predicting such an change in hearing loss progression will be highly challenging (especially without learning additional factors that *cause* hearing changes). On the other hand, to what degree such within-individual concept drift exists in the data is an empirical question. Fig 3 shows that the OLS model achieves an MSE score of 427 in this task. The mixed-effects regression and the mixture of regressions model are much more robust to the task change: the mixed-effects regression is still the best performing model, achieving an MSE score of 58 or a 7.6-decibel average prediction error; the mixture of regressions model approaches the accuracy of the mixed-effects regression as the number of clusters increases to six (the same number of clusters as in the data imputation task), where the MSE is 69 and the average error is 8.3 decibels. The estimated coefficients of regression predictors for each cluster is listed in Table 3.

**Table 3:** Correlation between audiogram response threshold and age as estimated by the mixed-effects regression (showing population-level effects only) and the mixture of regressions model in the future forecasting task. Numbers in parentheses indicate standard errors.

	*mixed-effects*	*mixture of regressions*					
		Cluster 1	Cluster 2	Cluster 3	Cluster 4	Cluster 5	Cluster 6
size	—	4,372	7,217	6,393	7,801	6,437	12,038
log(age)	0.82**	2.61***	0.56	-0.30	-0.32	-0.37	-1.37***
	(0.26)	(0.32)	(0.37)	(0.35)	(0.26)	(0.29)	(0.10)
gender(F)	0.94*	0.71	-3.09***	24.40***	8.53***	-35.69***	0.35***
	(0.40)	(0.37)	(0.53)	(0.53)	(0.35)	(0.41)	(0.10)
log(diag.)	0.51*	0.09	1.47***	0.83***	0.45***	0.52***	0.12**
	(0.06)	(0.15)	(0.16)	(0.14)	(0.09)	(0.10)	(0.04)
(intercept)	26.71***	61.41***	39.49***	28.3***	18.11***	53.35***	13.05***
	(0.63)	(0.79)	(0.91)	(0.84)	(0.64)	(0.67)	(0.22)

Note:

*p<0.05;

**p<0.01;

***p<0.001

These results suggest that, *on average*, there is very little within-individual concept drift in our data. Withholding the last data point of each PEF has a minimal effect on the performance of the mixed-effects model and the mixture of regressions model, where between-individual concept drift has already been taken into consideration. However, it is conceivable that within-individual concept drift is more likely for a distant future audiogram than a close one. If so, since the mixed-effects regression fits individual-level random slopes only to the observed data of that individual, it magnifies its susceptibility to concept drift due to within-individual variation, and therefore it is likely to be more inaccurate in predicting future observations that are further away. In contrast, the issue of within-individual concept drift should be mitigated for the mixture of regressions model, because it fits a slope of age to an entire latent cluster of PEFs. When predicting the withheld future observations, the mixture of regressions model will draw upon the average hearing change pattern within a cluster, which is much more robust against within-individual variations.

This hypothesis is tested by conducting an additional regression analysis, where the difference in the absolute error produced by the two models (i.e., |error by mixed-effects|—|error by mixture|) is regressed over the time gap between the last observed audiogram test and the withheld “future” observation. An additional control variable is also included: the actual observed threshold of the future audiogram (controlling for how the two models compare in predicting a high threshold). Confirming our hypothesis, the mixed-effects regression indeed tends to produce a larger error than the mixture of regressions model when the future observation is more distant (*β* = 0.10, *p* < 0.01). This finding suggests that the mixture of regressions model, rather than the mixed-effects regression, is potentially the more robust model against within-individual concept drift: although the mixed-effects model has produced the smallest *average* MSE in the current task, the magnitude of its prediction error increases faster than the mixture of regressions model the further into the future it tries to predict.

### Task 3: New patient generalization

The two prediction tasks we have investigated so far involve making predictions about withheld data points of previously observed individuals. In the third task, we specifically test the ability of the regression models to handle between-individual concept drift, by withholding all data points of a subset of randomly chosen PEFs as the test set (approximately 22% of the total data). To a trained regression model, the testing set thus represents audiograms of entirely “new” individual PEFs. This key design leads to a different form of between-individual concept drift than that of the data imputation task. In the data imputation task, the individual variations contributing to between-individual concept drift is fully observed *for each PEF* in the training data, so that the accuracy of prediction depends on how well the individual variations are captured. In the current task, however, the potential variations of the patients in the testing set are completely unobserved. Therefore, to handle this type of between-individual concept drift, a predictive model not only needs to capture the observed between-individual concept drift, but also generalizes to new individuals efficiently.

In evaluating the ability of each regression model to generalize to new individuals, we further impose the restriction that regression model parameters can only be estimated based on the training data (i.e. not on the test data). Audiogram results in the test set *cannot* be incrementally incorporated to the trained models to make predictions more accurate. In other words, although the new patient’s audiogram data is supplied to the trained model as input to make predictions, that data cannot be used to update the regression coefficients. This prediction task thus mimics a common clinical situation where a pre-trained predictive model must quickly generalize to a new patient, without having to continuously re-estimate the model for each new patient.

**Fig 4:**
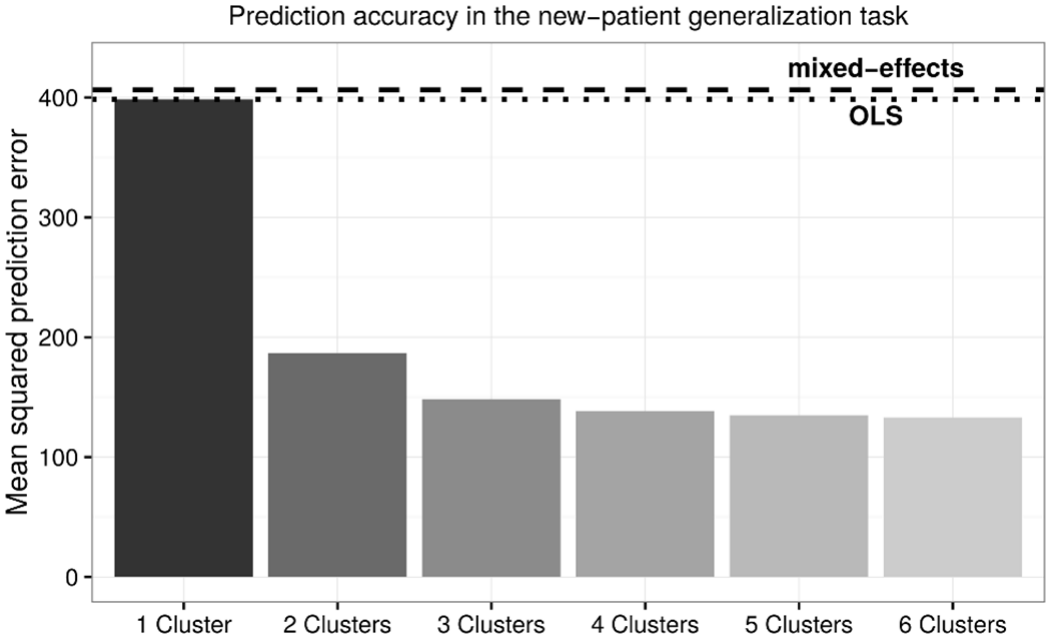
In the simulated new patient generalization task, the mixed-effects performed the worst among all models, reversing the pattern observed in the first two tasks. The mixture of regressions model achieved the lowest MSE with five clusters due to its robust generalization capabilities.

Fig 4 shows the prediction accuracy of all regression models in the current task. While an MSE of 398 is unsurprising for the OLS model (dotted line), a MSE of 406 for the mixed-effects regression model (dashed line) is surprisingly high given its superior performance in the previous two tasks. This dramatic increase in prediction error is due to the fact that the mixed-effects regression only fits random effects to observed and known individuals. Without re-training the entire model, the mixed-effects regression falls back to use the population-level fixed effects for making predictions when encountering a new individual. Therefore, at the prediction stage, the mixed-effects regression model is essentially reduced to the OLS model despite its complex mixed-effects structure.

Intriguingly, the mixture of regressions model continues to make relatively accurate predictions in the current task. Similar to the previous two tasks, its prediction error decreases as the number of latent clusters increases, reaching an asymptotic MSE of 133 (or a prediction error of 11.5 decibels) with six latent clusters. Regression coefficients for these six clusters are listed in Table 4. This notably better prediction accuracy is achieved via the latent representation of clusters inferred during the training phase: these latent clusters form the basis of performing further inferences about the probability of each new patient in the testing data belonging to the latent clusters (see Eq (7), which is then in turn used to optimally combine the predictions of different latent clusters about the audiogram thresholds for the new patients (see Eq (6)). In other words, the problem of between-individual concept drift is handled as a problem of latent categorization under the mixture of regressions model—the drift in the hearing change patterns of new patients is recognized as the recurrence of familiar patterns that have already been learned from the training data.

**Table 4:** Coefficients of regression predictors as estimated by the mixture of regressions model in the task of generalizing to new PEFs. Numbers in parentheses indicate standard errors.

size	*mixture of regressions*					
	Cluster 1	Cluster 2	Cluster 3	Cluster 4	Cluster 5	Cluster 6
	4,517	7,285	6,338	8,940	11,425	3,232
log(age)	3.18***	1.44***	-0.35	-0.58*	-1.09***	-1.96***
	(0.33)	(0.34)	(0.28)	(0.29)	(0.13)	(0.22)
gender(F)	-0.50	-10.89***	33.13***	-5.61	3.04***	-31.69***
	(0.35)	(0.43)	(0.36)	(0.39)	(0.20)	(0.43)
log(diag.)	-0.23	1.39***	0.68***	0.55***	0.19***	0.01
	(0.16)	(0.15)	(0.11)	(0.11)	(0.05)	(0.08)
(intercept)	66.61***	48.39***	17.84***	28.76***	10.21***	40.05***
	(0.26)	(0.33)	(0.30)	(0.25)	(0.19)	(0.14)

Note:

*p<0.05;

**p<0.01;

***p<0.001

There remains the challenging case where the hearing change pattern of a new patient has never been observed in the training data, namely, an event of concept drift that should result in the creation of a new latent cluster. Unfortunately, our current implementation of the mixture of regressions model does not recognize novel latent clusters during the prediction stage. Instead, it would categorize the patient into an existing latent cluster, resulting in suboptimal predictions for that new patient. The existence of such cases is likely the primary reason why the MSE of the mixture of regressions model is higher than in the previous two prediction tasks. A potential solution to this problem is discussed as part of the future directions for our work in the next section.

## Discussion

By comparing the prediction accuracy of three regression models in three prediction tasks, we have addressed the two questions as laid out at the beginning of the article. We have shown that the performance of a predictive model strongly depends on the goal of the prediction task. For the purpose of imputing randomly and uniformly distributed missing data, the mixed-effects model performed significantly better than either the ordinary least squares regression or the mixture of regressions model. However, if the goal of a prediction task is to extrapolate the further development of a patient’s condition based on observed history, the mixed-effects model, due to its tendency to overfit individual patterns, becomes less of an appropriate choice, especially for predicting long-term prognosis. Instead, the mixture of regressions model tends to perform better in this regard. Moreover, the mixture of regressions model is the only viable choice when the prediction goal is to generalize from observed patterns to new patients. Both the OLS model and the mixed-effects model generalized poorly, as neither of them has a prediction mechanism for making reasonable inferences about new patients.

We have also demonstrated that latent class modeling is a potentially effective strategy for dealing with concept drift in healthcare data. In our application of the mixture of regressions model, a probabilistic inference process was conducted so that individuals were categorized into different latent clusters, each of which was described by a unique set of regression coefficients. Given these clusters of distinct hearing change patterns, concept drift can then be modeled as the recurrence of familiar patterns. In general, such a strategy is particularly useful if the training data is relatively large in size, such that the inferred latent clusters will likely cover a significant portion of all possible patterns. In our opinion, the possibility of discovering *many* latent patterns is a key advantage of “big data” in biomedical informatics research. These latent patterns contribute greatly to the understanding of “naturally occurring” phenomena in the clinical process, which would have been difficult to conceive of in a hypothesis driven experiment. For instance, we have demonstrated that nearly half of the PEFs in our data are identified as exhibiting no hearing loss or even hearing improvement. The discovery of such patients not only helped predict the hearing change patterns of similar patients in the testing data, it has also raised interesting directions for domain experts to explore what factors could explain these unexpected hearing change patterns.

Although the primary intent of our work here is to tease apart the different types of prediction tasks in biomedicine, our findings may potentially contribute to the understanding of hearing loss progression as well. There have been so far an extremely limited number of studies that attempt to directly predict hearing thresholds in an audiogram test as we did. An early work by Hall and colleagues reported several regression-based methods for predicting hearing threshold in a setting that is similar to our imputation task, and found that in the best-case scenario, 53% of all test cases were predicted with an error margin of more than 10 decibels [28]. Our results thus compare favorably, although one should take into consideration the caveat that the underlying data differ significantly from ours. A relatively recent meta study modeled hearing loss progression as discrete severity states in an *observed* Markov model—that is, hearing change patterns are fitted as Markov chains with pre-defined hearing loss states [29]. The continuous nature of the regression predictor age in our methods thus represents a significant difference in the underlying assumption about the hearing loss problem: the progression of hearing loss (or improvement) is treated as a continuous event rather a discrete one. In reality, both continuous and discrete factors are probably influencing hearing changes, and more research in this area is warranted.

### Caveats and future directions

An important limitation of the mixture of regressions model in the current study is that the number of latent clusters must be specified manually, and once specified, it remains fixed. This represents both a practical problem, as it requires a trial-and-error stage for finding the optimal number of clusters, and a theoretical problem, as the fixed number of clusters restricts the model from adapting to potentially novel patterns. Advanced latent class models known as infinite mixture models, which optimally adapt the number of latent clusters to the data, have been widely studied in the machine learning community (e.g., [30, 31]). We plan to investigate whether additional benefits can be obtained by applying such methods to our data in future work.

Additionally, our research is partly motivated by the goal of implementing a predictive system of hearing loss progression that could be used in a real-world clinical setting for the pediatric patient population. Such a system would be most commonly required to perform prediction tasks that are similar to the future forecasting task and the new patient generalization task investigated here. For the goal of forecasting, our regression models, as simple as they are, performed reasonably well, achieving a prediction error of approximately 8 decibels on average. For the objective of new patient generalization, however, using age, gender, and the number of diagnoses as the only predictors is clearly insufficient and lead to a much larger prediction error of about 11.5 decibels on average. In general, a notable drawback of the current set of regression models is that they do not attempt to learn the medically relevant *causes* of hearing change patterns, but essentially try to *adapt* to the changing patterns as well as possible. Therefore, these models are not capable of understanding the underlying reasons for the hearing change pattern of an observed or new patient by referring to the patient’s overall medical condition. To address this problem, we are investigating the possibility of utilizing diagnosis results in a more sophisticated manner to capture the *causal* relations between medical conditions and hearing change patterns. Our ongoing efforts focus on examining whether patients discovered to belong to different latent clusters in our analysis are associated with certain unique sets of ICD9 codes. If successful, this approach may shed light on causes of different rates of hearing loss patterns in the sub-population of AudgenDB patients.
